# Health-care utilization for primary headache disorders in China: a population-based door-to-door survey

**DOI:** 10.1186/1129-2377-14-47

**Published:** 2013-06-03

**Authors:** Ruozhuo Liu, Shengyuan Yu, Mianwang He, Gang Zhao, Xiaosu Yang, Xiangyang Qiao, Jiachun Feng, Yannan Fang, Xiutang Cao, Timothy J Steiner

**Affiliations:** 1 Department of Neurology, Chinese PLA General Hospital, Beijing 100853, China; 2 Department of Neurology, The Fourth Military Medical University, Xian, Shaanxi Province, China; 3 Department of Neurology, Xiangya Hospital of Centre-south University, Changsha, Hunan Province, China; 4 Department of Neurology, Affiliated Huashan Hospital of Fudan University, Shanghai, China; 5 Department of Neurology, The First Hospital of Jilin University, Changchun, Jilin Province, China; 6 Department of Neurology, The First Affiliated Hospital of Sun Yat-sen University, Guangzhou, Guangdong Province, China; 7 Department of Health and Economics, Chinese PLA General Hospital, Beijing, China; 8 Department of Neuroscience, Norwegian University of Science and Technology, Trondheim, Norway; 9 Department of Neurology, Chinese PLA General Hospital, Fuxing Road 28 Haidian District, Beijing 100853, China

**Keywords:** China, Headache disorders, Migraine, Tension-type headache, Health-care utilization, Global campaign against headache

## Abstract

**Background:**

In order to know the *status quo* of health care for primary headache disorders in China, questions about headache consultation and diagnosis were included in a nationwide population-based survey initiated by *Lifting The Burden*: the Global Campaign against Headache.

**Methods:**

Throughout China, 5,041 unrelated respondents aged 18–65 years were randomly sampled from the general population and visited unannounced at their homes. After basic sociodemographic and headache diagnostic questions, respondents with headache answered further questions about health-care utilization in the previous year.

**Results:**

Significantly higher proportions of respondents with migraine (239/452; 52.9%) or headache on ≥15 days per month (23/48; 47.9%) had consulted a physician for headache than of those with tension-type headache (TTH) (218/531; 41.1%; P < 0.05). Multivariate analysis showed associations between disability and probability of consultation in those with migraine (mild vs. minimal: AOR 3.4, 95% CI: 1.6–7.4; moderate vs. minimal: 2.5, 1.2–5.4; severe vs. minimal: 3.9, 1.9–8.1) and between rural habitation and probability of consulting in those with TTH (AOR: 3.5; 95% CI: 1.9–6.3, P < 0.001). Married respondents with TTH were less likely than unmarried to have consulted (AOR: 0.26; 95% CI: 0.07–0.93; P = 0.038). About half of consultations (47.8–56.5%) for each of the headache disorders were at clinic level in the health system. Consultations in level-3 hospitals were relatively few for migraine (5.9%) but more likely for headache on ≥15 days/month (8.7%) and, surprisingly, for TTH (13.3%). Under-diagnosis and misdiagnosis were common in consulters. More than half with migraine (52.7%) or headache on ≥15 days/month (51.2%), and almost two thirds (63.7%) with TTH, reported no previous diagnosis. Consulters with migraine were as likely (13.8%) to have been diagnosed with “nervous headache” as with migraine. “Nervous headache” (9.8%) and “vascular headache” (7.6%) were the most likely diagnoses in those with TTH, of whom only 5.6% had previously been correctly diagnosed. These were also the most likely diagnoses (14.0% each) in consulters with headache on ≥15 days/month.

**Conclusions:**

This picture of the *status quo* shows limited reach of headache services in China, and high rates of under-diagnosis and misdiagnosis in those who achieve access to them. This is not a picture of an efficient or cost-effective response to major causes of public ill-health and disability.

## Background

Headache disorders are common and disabling throughout the world [1]. In the Global Burden of Disease survey 2010, tension-type headache (TTH) and migraine were identified as the second and third most prevalent disorders in the world, and migraine as the seventh highest specific cause of disability [2]. These, and a group of disorders characterized by headache on ≥15 days/month, have a major impact on public health and impose enormous economic costs on society [1-6]. Our recent door-to-door survey of the adult population of China showed that these disorders are common, burdensome and very costly in this country, and that health policy must take account of them [7].

How effectively do health services meet the needs of people with headache? Published accounts from western countries, notably the United States of America, report that a minority of people with migraine have received this diagnosis from a physician [8-12]; furthermore, of this minority, most do not receive effective therapy [12,13]. The global survey conducted by the World Health Organization (WHO) in collaboration with *Lifting The Burden*, as a project within the Global Campaign against Headache [14], found the political response to headache was inadequate in all countries, with health-care provision falling far below expectations based on need and on economics [15]. A consensus conference 15 years ago recommended studies to examine patterns of migraine diagnosis and treatment as the first step towards identifying and resolving barriers to its optimal management [16], but progress has been slow, as WHO’s survey lucidly and emphatically demonstrates [15].

In China there have been no such studies, and little is known about the *status quo* of consultations and diagnosis for people with headache in this country. We therefore built an enquiry into our recent population-based survey of headache disorders in China [7], conducted with support from *Lifting The Burden*[14]. Population-based surveys, when conducted well, offer a comprehensive and relatively unbiased overview of headache disorders. The additional enquiry reported here was designed primarily to assess the use of health resources for headache in China and, further, to identify possible systemic barriers to headache consultation, correct diagnosis and effective treatment.

## Methods

### Ethics

The study protocol was approved by the Chinese Ministry of Health and the Ethics Committee of the Chinese PLA General Hospital, Beijing.

### Data source

This was a secondary analysis of data from our previously reported nationwide epidemiological survey of primary headache disorders in the mainland of China [7]. In all regions of the country, households were selected using random-sampling software developed by a statistician (X-TC) according to the expanded programme on immunization (EPI) method of WHO [17]. These households were visited unannounced (“cold-calling”), and one adult respondent (aged 18–65 years) was chosen randomly from each. Full methodological details are described elsewhere [7,18]. In this way we created a sample reasonably representative of the Chinese adult population.

### Enquiry

Each participant answered a structured questionnaire. Sociodemographic questions covered gender, age, ethnicity (Han *versus* other), body-mass index (BMI) (graded as underweight, normal, overweight, obese or morbidly obese [19]), marital status, habitation (urban *versus* rural), educational attainment (illiterate, primary school, secondary school, high school, college degree or higher) and annual household income (in CNY: low < 9,600; middle 9,600–59,999; high ≥60,000). The diagnostic question set was validated for migraine and TTH [18]. Participants reporting headache in the preceding year were asked about attack frequency (days/month), attack duration (< 1 hour, 1–24 hours, >24 hours), headache intensity (10-cm visual analogue scale: 0 = no pain, 1–3 = mild pain, 4–7 = moderate pain and 8–10 = severe pain) and associated disability (HALT index [20]: 0–5 days lost in 3 months = minimal; 6–10 = mild; 11–20 = moderate; >20 = severe).

For the specific purposes of this enquiry, they were also asked about health-care utilization: consultations with physicians (in what health-care settings) and diagnoses received (if any). Health-care settings were classified as western medicine (WM) or traditional Chinese medicine (TCM) hospitals. The WM hospitals included community or rural, county or district, municipal and provincial hospitals. In conformity with this structure, we formulated a three-level model of health care for primary headache disorders: community or rural hospitals or clinics or county or district hospitals as level-1 hospitals, municipal hospitals as level-2 hospitals and provincial hospitals as level-3 hospitals.

### Statistical analysis

We looked for associations between sociodemographic factors or headache features and consultation status (consulted *versus* not consulted). We derived crude ratios of the proportion of consulters in each variable category to that of a reference category. We categorized previous headache diagnoses as “undiagnosed”, “migraine”, “tension-type headache”, “cluster headache”, “vascular headache”, “nervous headache” or “other”.

Statistical analyses were performed with SPSS 17.0 software. Continuous variables were summarized as means and standard deviations, and categorical variables as numbers and percentages. We used chi-squared to compare distributions of categorical variables between groups, and used Bonferroni correction to adjust the statistical results for multiple comparisons. We set statistical significance at P < 0.05. We first used binary logistic regression to estimate odds ratios (ORs) with 95% confidence intervals (CIs) for consulting, and adjusted for potential confounding variables (adjusted odds ratios: AORs), and then used multivariate logistic regression according to sociodemographic characteristics and headache features.

## Results

There were 5,041 participants, of whom 1,200 reported headache in previous year and 1060 participants met diagnostic criteria for migraine (n = 469), TTH (n = 543) or headache on ≥15 days/month (n = 48). Among these, 1,031 (452 migraine, 531 TTH, 48 headache on ≥15 days/month) answered the questions about consultation. Fewer than half (46.6%) of the participants with headache reported at least one consultation with any physician for their headache in the year prior to the interview. Those with migraine (52.9%) or headache on ≥15 days/month (47.9%) were significantly more likely to have consulted than those with TTH (41.1%; P < 0.05).

Univariate analysis (Table 1) suggested females with migraine (55.4%) were somewhat more likely than males with migraine (47.6%) to have consulted; this difference was not significant (P = 0.122). No gender difference was seen among those with TTH. Among those with migraine, there was a shallow and non-significant, but nonetheless consistent, tendency of increasing probability of consulting with advancing age (from 44.4% in those under 30 years to 55.4% in those over 60). No such tendency was seen for TTH. Marital status had no obvious effect in this analysis, but the numbers of respondents who were unmarried (single, divorced or widowed) were very low. Ethnicity (non-Han) increased the probability of consulting (by a factor of 1.3 in those with migraine), but again numbers were low and this was not significant (P = 0.119). However, rural habitation significantly increased the probability of consulting (urban migraine: 44.4%; rural migraine: 57.6%; P = 0.007; urban TTH 28.4%; rural TTH: 49.2%; P < 0.001). Educational attainment was inversely related to probability of consulting for migraine but not for TTH. Household income had no clear effect; neither did BMI.

**Table 1:** Proportions of respondents reporting consultation for headache in the preceding year, according to diagnosis and socio-demographic characteristics

	**Migraine**					**TTH**				
	**Total (N)**	**Proportion consulted**				**Total (N)**	**Proportion consulted**			
		**n (%)**	**Crude ratio**	**P**	**Bonferroni adjusted P**		**n (%)**	**Crude ratio**	**P**	**Bonferroni adjusted P**
**Gender**										
Male	145	69 (47.6)	1.000			194	77 (39.7)	1.000		
Female	307	170 (55.4)	1.164	0.122		337	141 (41.8)	1.054	0.628	
**Age (years)**										
18–29	27	12 (44.4)	1.000			56	22 (39.3)	1.000		
30–39	99	49 (49.5)	1.114	0.642	1.000	101	38 (37.6)	0.958	0.837	1.000
40–49	150	81 (54.0)	1.215	0.362	1.000	159	74 (46.5)	1.185	0.348	1.000
50–59	111	61 (55.0)	1.236	0.328	1.000	133	55 (41.4)	1.053	0.792	1.000
60–65	65	36 (55.4)	1.246	0.340	1.000	82	29 (35.4)	0.9..	0.640	1.000
**Ethnicity**										
Han	427	222 (52.0)	1.000			492	198 (40.2)	1.000		
Other	25	17 (68.0)	1.308	0.119		39	20 (51.2)	1.274	0.180	
**Marital status**										
Single	13	5 (38.5)	1.000			34	16 (47.1)	1.000		
Married	422	225 (53.3)	1.386	0.297	1.000	472	190 (40.3)	0.855	0.437	1.000
Divorced	7	4 (57.1)	1.486	0.427	1.000	10	4 (40.0)	0.850	0.694	1.000
Widow	10	5 (50.0)	1.300	0.581	1.000	15	8 (53.3)	1.133	0.686	1.000
**Habitation**										
Urban	162	72 (44.4)	1.000			208	59 (28.4)	1.000		
Rural	290	167 (57.6)	1.296	0.007		323	159 (49.2)	1.735	<0.001	
**Educational attainment**										
Illiterate	46	31 (67.4)	1.863			56	25 (44.6)	1.190		
Primary school	138	81 (58.7)	1.623	0.003	0.012	161	63 (39.1)	1.043	0.132	0.528
Secondary school	140	77 (55.0)	1.521	0.008	0.032	155	73 (47.1)	1.256	0.270	1.000
High school	81	33 (40.7)	1.126	0.027	0.108	95	37 (38.9)	1.039	0.032	0.128
College level or higher	47	17 (36.2)	1.000	0.610	1.000	64	24 (31.2)	1.000	0.322	1.000
**Annual household income (CNY)**										
Low	124	68 (54.8)	1.000			129	62 (48.1)	1.000		
Middle	289	148 (51.2)	0.934	0.499	0.998	340	135 (39.7)	0.826	0.102	0.204
High	33	19 (57.6)	1.050	0.779	1.000	45	15 (33.3)	0.693	0.089	0.178

Table 2 shows the influence of headache characteristics. Univariate analysis found that, among those with migraine, headache severity and frequency were each directly related to probability of consulting. The same influences were seen in TTH, but smaller and non-significant. Attack duration had no clear influence. In both disorders, higher disability (measured as lost productive time using the HALT index [20]) was also associated with greater probability of consulting, but this was almost entirely explained by a large step-up between minimal and mild disability.

**Table 2:** Proportions of respondents reporting consultation for headache in the preceding year, according to diagnosis and headache characteristics

	**Migraine**					**TTH**				
	**Total (N)**	**Proportion consulted**				**Total (N)**	**Proportion consulted**			
		**n (%)**	**Crude ratio**	**P**	**Bonferroni adjusted P**		**n (%)**	**Crude ratio**	**P**	**Bonferroni adjusted P**
**Headache intensity**										
Mild	62	22 (35.5)	1.000			161	58 (36.0)	1.000		
Moderate	281	154 (54.8)	1.544	0.007	0.014	328	143 (43.6)	1.210	0.110	0.220
Severe	107	63 (58.9)	1.659	0.004	0.008	40	16 (40.0)	1.110	0.641	1.000
**Headache frequency (days/month)**										
≥15	41	27 (65.9)	1.491	0.015	0.030	25	12 (48.0)	1.249	0.354	0.708
1–14	235	127 (54.0)	1.224	0.057	1.104	243	100 (41.2)	1.071	0.552	1.000
<1	154	68 (44.2)	1.000			216	83 (38.4)	1.000		
**Disability (HALT grade)**										
Minimal	191	70 (36.6)	1.000			297	100 (33.7)	1.000		
Mild	54	36 (66.7)	1.819	<0.001	<0.001	41	21 (51.2)	1.521	0.028	0.084
Moderate	57	36 (63.2)	1.723	<0.001	<0.001	36	20 (55.6)	1.650	0.010	0.030
Severe	92	67 (72.8)	1.987	<0.001	<0.001	65	35 (53.8)	1.599	0.002	0.006

Multivariate analysis (Table 3) confirmed the association between disability and probability of consulting in those with migraine (mild vs. minimal: AOR 3.4, 95% CI 1.6–7.4; moderate vs. minimal: 2.5, 1.2–5.4; severe vs. minimal: 3.9, 1.9–8.1) but not in those with TTH (mild vs. minimal: AOR 1.6, 95% CI: 0.72–3.6; moderate vs. minimal: 1.8, 0.73–4.3; severe vs. minimal: 1.5, 0.69–3.2). In TTH it also confirmed the association between rural habitation and probability of consulting (AOR: 3.5; 95% CI: 1.9–6.3, P < 0.001), and additionally showed that married respondents were less likely to have consulted (AOR: 0.26; 95% CI: 0.07–0.93, P = 0.038).

**Table 3:** Predictors of consultation for headache in the preceding year according to multivariate adjusted binary logistic regression

	**Migraine**		**TTH**	
	**Adjusted odds ratio (95% CI)**	**P**	**Adjusted odds ratio (95% CI)**	**P**
**Marital status**				
Married *versus* single, divorced or widowed	0.64 (0.15, 2.8)	0.557	0.26 (0.07, 0.93)	0.038
**Habitation**				
Rural *versus* urban	1.2 (0.65, 2.1)	0.609	3.5 (1.9, 6.3)	< 0.001
**Disability (HALT grade)**				
Mild *versus* minimal	3.4 (1.6, 7.4)	0.002	1.6 (0.72, 3.6)	0.243
Moderate *versus* minimal	2.5 (1.2, 5.4)	0.020	1.8 (0.73, 4.3)	0.210
Severe *versus* minimal	3.9 (1.9, 8.1)	< 0.001	1.5 (0.69, 3.2)	0.312

For all of these analyses, numbers with headache on ≥15 days/month were too low.

Table 4 shows that about half of consultations (47.8–56.5%) for each of the headache disorders were at clinic level in the health system. Of consultations in hospitals, about half (23.8–30.4% of all consultations) were at level 1. In other words, about three quarters of consultations (71.9%) were at the bottom levels. Consultations in level-3 hospitals were relatively few for migraine (5.9%) but more likely for headache on ≥15 days/month (8.7%) and, surprisingly, for TTH (13.3%). Consultations at TCM hospitals were undertaken by 7.9% of respondents with migraine, 6.9% with TTH and none with headache on ≥15 days/month.

**Table 4:** Level and setting of consultation for headache in the preceding year

	**Migraine**	**TTH**	**Headache on ≥15 days/month**
	**n (%)**	**n (%)**	**n (%)**
**Level or setting**			
Clinics	135 (56.5)	112 (51.4)	11 (47.8)
Level-1 hospitals	57 (23.8)	64 (29.4)	7 (30.4)
Level-2 hospitals	46 (19.2)	38 (17.4)	6 (26.1)
Level-3 hospitals	14 (5.9)	29 (13.3)	2 (8.7)
Traditional Chinese medicine hospitals	19 (7.9)	15 (6.9)	0 (0.0)
Others	8 (3.3)	5 (2.3)	0 (0.0)
**Total**	239 (100.0)	218 (100.0)	23 (100.0)

Under-diagnosis and misdiagnosis were common in consulters. More than half with migraine (52.7%) or headache on ≥15 days/month (51.2%), and almost two thirds (63.7%) with TTH, reported no previous diagnosis (Table 5). Consulters with migraine were as likely (13.8%) to have been diagnosed with “nervous headache” as with migraine; otherwise the most common previous diagnosis was “vascular headache” (9.4%). “Nervous headache” (9.8%) and “vascular headache” (7.6%) were the most likely diagnoses in consulters with TTH, of whom only 5.6% had previously been correctly diagnosed. These were also the most likely diagnoses (14.0% each) in consulters with headache on ≥15 days/month.

**Table 5:** Previous diagnoses in respondents reporting consultation for headache in the preceding year according to survey diagnosis

**Survey diagnosis**	**Migraine**	**TTH**	**Headache on ≥15 days/month**
	**n (%)**	**n (%)**	**n (%)**
**Previous diagnosis**			
Undiagnosed	218 (52.7)	319 (63.7)	22 (51.2)
Migraine	57 (13.8)	13 (2.6)	1 (2.3)
TTH	3 (0.7)	28 (5.6)	0 (0.0)
Cluster headache	4 (1.0)	2 (0.4)	0 (0.0)
Vascular headache	39 (9.4)	38 (7.6)	6 (14.0)
Nervous headache	57 (13.8)	49 (9.8)	6 (14.0)
Others	36 (8.7)	52 (10.4)	8 (18.6)
**Total**	414 (100.0)	501 (100.0)	43 (100.0)

## Discussion

This first nationwide population-based survey of primary headache disorders in China provides much-needed insights into the *status quo* of health-service utilization and diagnosis in this very large country.

A striking finding of the study is that a majority (53.4%) of people with an active headache disorder had not consulted a physician for it in the year prior to the survey. Nevertheless, this is in complete accord with studies from a number of other countries in Europe and North and South America [21-31]. Perhaps it is the case that the proportion of consulters (46.6%) is even a little higher in China than in western countries. This may not have so much to do with the availability of medical resources as with the barriers to access that exist in different countries, and the differences in care-seeking behaviour among culturally-different populations. Many European medical systems require referral from a primary-care physician in order to seek the advice of a specialist and, in published surveys, 5–15% of people with headache have done so [26,32,33]. Mainland China has a system allowing prospective patients to choose any level of medical setting at which to seek diagnosis and treatment for headache. In our survey, 28.1% of respondents who consulted did so at levels 2 or 3 of the hospital system, which represents 13.1% of everyone reporting headache. There is a continuing debate about the efficiency and cost-effectiveness of free access of this sort rather than controlled access according to need [15,34].

It may be that these levels of consultation-below 50%-do, in fact, reflect need; that is to say, those who are little disabled by their headaches do not *need* professional health care, but can manage themselves adequately, using over-the-counter medications if necessary. The crucial question is whether the minority who consult are those most likely to benefit. From this perspective, the predictors of consultation are of interest. According to univariate analysis, educational attainment was inversely related to probability of consulting for migraine but not for TTH. Household income had no clear effect. Rural habitation significantly increased the probability of consulting, although this influence survived multivariate analysis only for TTH. Better education is likely to promote self-efficacy and better control over headache attacks with the help of knowledge gleaned from books and the internet. Those who are poorly educated, lacking access to such knowledge, may have greater fear of headache as a symptom, and what it might mean, and this would presumably encourage consultation. The influence of rural habitation is not easily explained; it is speculative to suggest that the rural lifestyle allows more free time to visit a physician. As expected, headache severity and frequency were both positive predictors of consultation, especially for migraine, as was headache-attributed lost productive time. These may be regarded as key indicators of need for health care, suggesting that resources in China are to some extent directed towards those most likely to benefit, but it has to be said that these influences were not strong.

Beyond the issue of access is the quality of care delivered to those who do consult. Our survey was not able to delve deeply into this, because of lack of standards for comparison, but we could consider diagnostic accuracy, having in mind the certain fact that this is a prerequisite for good care [35]. It is the case that under-diagnosis and to some extent misdiagnosis of headache are worldwide concerns [15]. The picture in China that our survey painted is no different, and not an encouraging one. An accurate diagnosis of migraine was recorded in only 13.8% of consulting respondents with this disorder-the same proportion as were given the non-diagnosis of “nervous headache”. Over half had been given no diagnosis. For TTH the situation was substantially worse, with just 5.6% of consulting respondents diagnosed correctly while 63.7% were undiagnosed. The non-diagnoses of “vascular headache” and “nervous headache” were common across all disorders, and accounted for 28.0% of those with headache on ≥15 days/month. Clearly, the international classification of headache disorders (ICHD-II) [36], almost universally adopted elsewhere, is not widely accepted or used in most hospitals in China. Since management guidelines are fundamentally diagnosis-based, this finding does not raise expectations of high-quality or effective heath care for headache in this country.

Three clear calls arise from this study, directed towards improving awareness of primary headache disorders among people affected by them and among medical professionals in China, so that these disabling disorders might be regarded with greater respect, and better treated. The first call is for public education, explaining the high burden of headache disorders, their biological basis and the effective treatments that are potentially available for them if sought. This education should promote proper and considerate use of health care. It should allay groundless fears of serious disease, such as brain tumour, which provoke many unnecessary consultations at specialist level. The second call is for professional education, bringing ICHD-II [36] into clinical practice in China so that diagnosis-and management-can come into line with international standards [35]. This call for professional education in headache echoes a similar call, directed at all countries, by WHO and *Lifting The Burden* following their global survey [15]. The third call is to politicians, to channel the resources these initiatives require. Ultimately, in view of the very high lost-productivity costs of headache in China [7], this would surely be cost-saving [15].

### Strengths and limitations of the study

There are limitations to our study, but we do not believe they have the effect of negating the key findings. First, we enquired into medical consultations only within the past year. If a headache patient was adequately treated in the year before last, or even earlier, consultation this year might not have been necessary. In theory this could be one reason for the low consultation rate but, given the evident deficiencies in diagnosis, it is highly unlikely that this was the case. In fact, some recall bias is to be expected in reporting consultations over a year. If there was, it is more likely that it resulted in overestimates. People are unlikely to forget the fact of having consulted, but may not remember exactly when they did so. Since people tend to recall past time as shorter in duration than reality, they may include events from a longer period than the specified year. Second, respondents may not have recalled diagnoses previously given, but we would argue that a forgotten diagnosis had not been adequately or effectively imparted. Third, we could not make complete allowance for the use of TCM hospitals, which may have reduced dependence on and consultation rates at WM hospitals. But the number using TCM hospitals was small (n = 34; 3.3% of those reporting headache), so this could not have had more than a negligible effect.

On the other hand, this study used well-established and validated EPI methods [17] in a population-based nationwide study. The large sample of 5,041 participants was representative of the general population of mainland China, aged 18–65 years, for gender, age distribution, socioeconomic status, type of habitation and region of residence [7,18].

## Conclusion

This epidemiological survey, performed in order to depict the *status quo* of consultations and diagnosis for people with headache in mainland China, has demonstrated limited reach of headache services in this country and high rates of under-diagnosis and misdiagnosis in those who achieve access to them. This is not a picture of an efficient or cost-effective response to major causes not only of public ill-health and disability but also of an enormous societal financial burden from lost productivity [7]. Public and professional educational initiatives are needed to redress these deficiencies, and these require political support. In this respect, China is little different from other countries [8-12,15].

## Abbreviations

TTH: Tension-type headache; WHO: The World Health Organization; WM: Western medicine; TCM: Traditional Chinese medicine; ICHD-II: The international classification of headache disorders.

## Competing interests

All authors declare there are non-financial competing interests (political, personal, religious, ideological, academic, intellectual, commercial or any other) in relation to this manuscript.

## Authors’ contributions

M.D. RL, MH, GZ, XY, XQ, JF and YF carried out the studies, participated in the survey. And RL drafted the manuscript. Professor SY, the PI of this survey, and Dr. MH and Dr. XC participated in the design of the study and performed the statistical analysis. Professor TJS conceived of the study, and participated in its design and coordination and helped to draft the manuscript. All authors read and approved the final manuscript.
